# Relevance of body weight adaptation and modern obesity‐defining parameters in the analysis of isokinetic trunk strength in people with obesity – A retrospective analysis

**DOI:** 10.1111/cob.12736

**Published:** 2025-01-23

**Authors:** Daniel Geissler, Andreas Lison, Christoph Schulze

**Affiliations:** ^1^ Orthopädische Klinik und Poliklinik Rostock Germany; ^2^ Zentrum für Sportmedizin der Bundeswehr Warendorf Germany; ^3^ Universitätsinstitut für Physikalische Medizin und Rehabilitation Universitätsklinikum Salzburg Salzburger Landeskliniken Salzburg Austria

**Keywords:** body‐weight adaption, obesity, trunk strength, waist‐to‐height ratio

## Abstract

Pathologic values of body mass index (BMI), body weight, and waist circumference correlate with higher absolute and lower relative trunk strength. Whether waist‐to‐height ratio (WHtR) is appropriate for showing trunk strength differences in people with obesity and whether a continuous linear relationship exists between the increase in obesity and trunk strength is unknown. This retrospective cross‐sectional study included 1174 subjects (1114 men and 60 women). Measured values included body weight, height, waist circumference, WHtR, BMI, and both absolute and body weight‐adapted trunk flexor/extensor strength. Statistical analyses included *t*‐tests, Welch tests, Pearson correlations, mixed‐linear, and nonlinear regression analyses. Positive correlations with absolute trunk strength were found in subjects without obesity for all anthropometric parameters except WHtR. Weaker positive and partly negative correlation and linear regression coefficients were found in subjects with obesity. Nonlinear relationships were found between age, BMI, WHtR, and absolute respective body weight‐adapted trunk strength. The relationship between obesity‐defining measures/ indices and trunk strength is non‐linear. Increasing BMI, waist circumference, or WHtR above cut‐off values known from cardiovascular research is linked to a decrease or weaker increase in trunk strength. Body weight adaptation is recommended to avoid misinterpretation of apparently good absolute trunk strength values in people with obesity.

**What is already known about this subject**

- Higher weight, height, BMI (body mass index), and waist‐to‐height ratio (WHtR) are connected to higher absolute trunk strength.
- Higher BMI is connected to lower body weight‐adapted trunk strength.

**What the study adds**

- The relationship between BMI, waist circumference, WHtR, and absolute as well as relative trunk strength is nonlinear and mostly parabolic shaped.
- Pre‐existing cut‐off values of BMI and WtHR suit well for demonstrating trunk strength differences between individuals with and without obesity.
- To avoid misinterpretation of apparently good absolute trunk strength values in people with obesity, body weight adaptation is recommended.

## 1 INTRODUCTION

Obesity causes a significant proportion of the overall disease burden all over the world and is associated with a number of comorbidities.^1^ Among these are the increased incidence of low back pain as well as musculoskeletal disorders.^2^, ^3^ Both obesity and back pain are a burden on healthcare systems and result in an increased number of hospitalizations and operations, such as endoprosthetic procedures and intervertebral disc operations.^4^, ^5^, ^6^ Several factors were identified as possible reasons of pain in obese people. These include chronic inflammatory immune reaction and sensibilization of the nociceptors with increased cortisol secretion and a depressive mood alteration with increased vulnerability to pain,^7^ biomechanical effects consisting of increased shear forces on the trunk^8^, ^9^ and a change of body posture with a ventrocaudal shift of the centre of mass,^10^ as well as faster overall fatigue with lower anaerobic and aerobic capacity.^11^, ^12^ Reduced trunk strength in obese people resulting from a lack of exercise is an important factor in the multifactorial pathogenesis of back pain.^2^

Studies comparing absolute trunk strength between populations with or without obesity have been inconsistent so far. Both lower^13^ as well as higher^14^, ^15^, ^16^ absolute trunk strength values in obese people have been described. Studies that included body weight‐adapted muscle strength were able to show lower relative muscle strength in muscles of shoulder and knee in obese people.^15^, ^17^, ^18^ Other studies quantified the influences of obesity‐defining variables by correlation or regression analyses.^14^, ^19^, ^20^ For this purpose, correlation or regression analyses with trunk strength as the dependent variable and anthropometric factors as the independent variables were performed. Anthropometric variables in these specific studies were body weight and BMI. A correlation was observed between higher weight or BMI and higher absolute and lower body weight‐adapted trunk strength.^19^, ^20^ This contrast in absolute and body weight‐adapted trunk strength in obese people may be explained by the increased load on the trunk resulting in training effects among greater absolute muscle mass.^20^ Park et al. observed a lower body weight‐adapted trunk extensor strength in patients with low back pain and significant correlations between greater BMI and lower body weight‐adapted trunk extension and flexion strength.^13^ Meanwhile, waist circumference and waist‐to‐height ratio (WHtR) are established in the diagnosis of obesity. However, there are few studies that investigated the relationship between waist circumference and trunk strength. There is limited data regarding the relationship between WHtR and trunk strength. Individuals with the highest BMI and waist circumference had the worst results in the ‘static back extension test’ without significant results in correlation and regression analyses.^21^ Poor results in a 6‐item fitness test for firefighters, including isometric abdominal muscle testing, correlated with higher BMI and waist circumference, but without significant correlations between the abdominal muscle strength and BMI and waist circumference.^22^ WHtR correlated with higher flexion and extension trunk strength,^23^ whereas waist‐to‐hip ratio (WHR) correlated with lower body weight‐adapted trunk extension strength.^13^ Further, there are still no standardized WHtR cut‐off values for abdominal obesity. Previous studies focused primarily on the increase in cardiovascular risk and define obesity starting from WHtR values of 0.5–0.6.^24^ Schneider et al. proposed age‐related classifications of normal and pathological WHtR in a study regarding risk stratification of LDL cholesterol elevations: 18–39 years WHtR <0.5, 40–49 years WHtR <0.55 and >50 years WHtR <0.6.^25^, ^26^

With regard to the complex relationship between trunk strength and obesity, it is the question whether there is a suitable discriminating variable that indicates obesity and simultaneously allows the analysis of differences in trunk strength. The aim of this study was to identify the best cut‐off value for obesity‐specific trunk strength differences using BMI, waist circumference and WHtR in order to be able to give advice to obese patients regarding the improvement in trunk strength. In this context, the study was also intended to investigate how a pathologically increased WHtR affects body weight‐adapted trunk strength.

## 2 MATERIALS AND METHODS

### 2.1 Subjects

Data were collected from 1174 healthy subjects (1114 male and 60 female) that underwent both anthropometric and isokinetic trunk strength measurements from 2008 to 2018 during military medical fitness examinations. The collective consisted of German military personnel which led to a relatively low number of female individuals. Due to repeated measurements during the observation period from 2008 to 2018, a total of 3180 measurements were recorded. Group classification was based on the discriminating variables BMI, waist circumference, WHtR and age‐standardized WHtR. According to WHO recommendations, BMI >30 kg/m^2^ indicates obesity.^24^ Waist circumference of >102 cm in men and >88 cm in women results in an increased general morbidity risk and indicates abdominal obesity.^24^ A cut‐off value of 0.55 was set for the WHtR due to pre‐analyses of the data with strong trunk strength differences around 0.55. For the age‐standardized WHtR, we applied the cut‐off values according to Schneider et al.^25^ A total of 8 groups were defined, splitting the collective in individuals with or without obesity using the four different parameters BMI, waist circumference, WHtR and age‐standardized WHtR. Descriptive data including group sizes are shown in Table 1. All procedures performed were in accordance with the ethical standards of the institutional and national research committee and with the 1964 Helsinki Declaration and its later amendments. The institutional ethics board approved the conduct of this study (AZ: A2019‐0123).

**TABLE 1:** Anthropometric composition of the subjects in the groups with or without obesity.

Parameter	BMI normal (*n* = 2890)	BMI pathological (*n* = 290)	*t*‐test	WC normal (*n* = 2769)	WC pathological (*n* = 359)	*t*‐test	WHtR normal (*n* = 2711)	WHtR pathological (*n* = 417)	*t*‐test	Age‐related WHtR normal (*n* = 1778)	Age‐related WHtR pathological (*n* = 649)	*t*‐test
Sex (m/f)	981/57	170/3		942/50	222/12		923/53	265/5		796/51	342/8	
Age (years)	37.87	40.42	<0.001	37.27	44.65	<0.001	37.01	44.71	<0.001	36.89	34.69	<0.001
±SD	(±9.86)	(±9.74)	*	(±9.35)	(±11.34)	**	(±9.27)	(±11.13)	**	(±11.46)	(±7.77)	**
Height (cm)	180.73	181.27	0.13	180.65	182.09	<0.001	180.93	180.04	0.012	181.15	180.26	<0.001
±SD	(±6.88)	(±6.92)	*	(±6.8)	(±7.46)	*	(±6.91)	(±6.72)	*	(±7.10)	(±6.99)	*
Weight (kg)	83.57	108.16	<0.001	83.51	104.71	<0.001	83.54	101.53	<0.001	82.54	94.12	0.006
±SD	(±9.69)	(±11.86)	**	(±9.75)	(±13.3)	**	(±10.03)	(±13.83)	**	(±10.12)	(±13.1)	**
BMI (kg/m^2^)	25.51	32.87	<0.001	25.4	31.45	<0.001	25.45	31.16	<0.001	25.08	28.86	<0.001
±SD	(±2.08)	(±2.88)	**	(±2.28)	(±3.61)	**	(±2.11)	(±3.50)	**	(±2.14)	(±3.29)	**
WC (cm)	87.63	108.4	<0.001	87.12	108.86	<0.001	86.9	107.34	<0.001	85.86	98.36	<0.001
±SD	(±7.49)	(±9.42)	**	(±6.76)	(±8.08)	**	(±6.68)	(±8.24)	**	(±7.57)	(±9.26)	**
WHtR	0.49	0.6	<0.001	0.48	0.6	<0.001	0.48	0.6	<0.001	0.47	0.55	<0.001
±SD	(±0.04)	(±0.05)	**	(±0.04)	(±0.45)	**	(±0.03)	(±0.04)	**	(±0.04)	(±0.05)	**

*Note*: Age‐related WtHR pathological = age 18–39 years ≥0.50, 40–49 years ≥0.55, ≥50 years ≥0.60; Age related WtHR normal = age 18–39 years <0.50, 40–49 years <0.55, ≥50 years <0.60; BMI normal = <30 kg/m^2^; BMI pathological = >30 kg/m^2^; WC normal = <102 cm in men and <88 cm in women; WC pathological = ≥102 cm in men and ≥88 cm in women; WHtR normal = <0.55; WHtR pathological = ≥0.55; *t*‐test, significance of *t*‐test is displayed to demonstrate significant group differences between groups with or without obesity (*in case of variance homogeneity *t*‐test was used, **in case of variance heterogeneity welch‐test was used).

Abbreviations: BMI, body mass index; SD, standard deviation; WC, waist circumference; WtHR, waist‐to‐height ratio.

### 2.2 Anthropometric measurements

All anthropometric measurements (age, height, weight and waist circumference) were taken at the same time as isokinetic measurements. Waist circumference measurements were performed in accordance with WHO recommendations in a line parallel to the ground at the halfway point between the iliac crest and the lower costal margin.^24^ The indices BMI (body weight in kilograms divided by the squared height in meters) and WHtR (waist circumference in meters divided by height in meters) were calculated.

### 2.3 Isokinetic trunk strength measurements

The IsoMed2000 device (D.&R. Ferstl, Hemau, Germany) was used for isokinetic measurement of trunk flexion and extension strength. A gravity compensation feature was integrated in this device.^27^, ^28^ After a 10‐min warm‐up on a bicycle ergometer, the subjects were seated onto the machine and measurements were performed under supervision of an experienced instructor, encouraging the subjects to perform at maximum effort.^23^, ^29^ The procedure consisted of 10 repetitions at an angular velocity of 90° s^−1^.^27^, ^28^ Range of motion was −24° to +22°, starting from 90° flexion at the hip joint. All parameters were measured separately for trunk extension and flexion. Following measured parameters are termed as absolute trunk strength parameters: peak trunk extension torque and peak trunk flexion torque (best out of 10 repetitions in Newton meter). Body weight‐adapted parameters were calculated for flexion and extension using the actual body weight. There was no control group or condition to calculate the test quality, but rather 24 subjects who were examined twice over a period of 0–2 days. Based on this data, coefficients of variation (CV) and intraclass correlation coefficients (ICC type (1, k) according to Shrout and Fleiss^30^) were calculated. The VC for peak torque was 0.075 in trunk flexion and 0.134 in trunk extension. The ICC for trunk flexion (0.819) and trunk extension (0.875) were within a very good range according to Cicchetti.^31^

### 2.4 Statistical analyses

SPSS version 24 software (IBM, Armonk, New York, USA) was used to analyse the data. Visual analysis of normal distribution using histograms and Q–Q plots for large samples is superior to conventional normal distribution tests (i.e., Shapiro–Wilk test), therefore visual analysis took place.^32^ Normal distribution analyses showed a lack of normal distribution for height, body weight, BMI, WHtR, peak torque of trunk flexion and trunk extension in the 4 obesity groups. For age and gender, there was also no normal distribution, as the sample consisted mainly of young male subjects. To test for group differences within the obesity/ non‐obesity groups, both two‐sided *t*‐test and Welch‐test were used. Despite the lack of normal distribution in some cases, we used *t*‐tests according to the robustness of the *t*‐tests observed by Rasch and Guiard.^33^ Levene's test was used to test for variance homogeneity. Variance homogeneity was assumed at *p* > .05. In case of missing variance homogeneity, Welch's test was used, which is more robust and provides comparable results.^34^ In the next step, trunk strength variables on one hand and anthropometric measurement variables on the other hand were correlated using Pearson product–moment correlation to calculate the Pearson correlation coefficient (*r*). Correlation coefficients in the different groups were compared afterwards. The strength of correlation was interpreted according to Cohen.^35^ In addition, the data underwent mixed linear regression analyses for each of the specific isokinetic parameters. Residuals, respectively error terms, were then visually checked for normal distribution via histogram and Q–Q plot. To detect possible non‐linear correlations, the data also underwent non‐linear regression analyses using the open‐source statistical program ‘R’ (version 4.2.2, https://r-project.org, Copyright© 2021–2023 R Core Team). The package ‘gamm4’ was used to conduct generalized additive mixed regression analyses with isokinetic trunk strength as dependent variable. Age, waist circumference, WHtR, BMI and gender were included as fixed effects and the intraindividual measurement repetitions as random effects. Smoothing was used for BMI, age, waist circumference and WHtR. With smoothing, nonlinear effects are allowed and their functional form is estimated step‐by‐step from the data by dividing them into smaller sub‐formulas (splines). Since anthropometric factors naturally interact with each other, multicollinearity is likely. Careful and stepwise modelling was performed to eliminate this. Once BMI and WHtR were included in the models, despite known significant correlations, some gamm4‐models were not significant. For this reason, and because both BMI and WHtR are calculated out of the anthropometric factor height, we decided to use the following model constellation without WHtR but with waist circumference, avoiding multicollinearity by the indirect duplication of height.

The following formulas were used to evaluate the connection between trunk strength and age, BMI, waist circumference and WHtR in the gamm4‐models. ‘s’ stands here for smoothing of the parameter and ‘random=ID’ for the effect of repeated intraindividual measurements:

$$\begin{array}{l} \text{trunk strength}\sim \text{gender}+s\left(\mathrm{age}\right)+s\left(\mathrm{BMI}\right)+s\text{(waist circumference)}\\ \;+\;\text{random}\\ \;=\mathrm{ID}. \end{array}$$

One model describing the connection between BMI and body weight‐adapted trunk extensor strength was analysed using an alternative model constellation, where waist circumference (WC) and WHtR were included due to a lack of statistical significance.

To be able to provide conclusions about WHtR, we have included the WHtR in a separate model without BMI and without WC:

$$\text{trunk strength}\sim \text{gender}+s\;\left(\mathrm{age}\right)+s\;\left(\text{WHtR}\right)+\text{random}=\mathrm{ID}.$$

By comparison of the ‘effective degrees of freedom’, the non‐linear models were compared with the linear models. Lower ‘effective degrees of freedom’ indicate a more linear relationship, while higher ‘effective degrees of freedom’ indicate a higher ‘wiggliness’ meaning higher non‐linearity. The evaluation of the non‐linear regressions included optical analysis of the graphical output, significance and the optical normal distribution analyses of the residuals. Significance levels for all correlation and regression analyses were set at *p* < .05.

## 3 RESULTS

### 3.1 Results of anthropometric measurements

A total of 4 discrimination parameters were used to differentiate between subjects with and without obesity. From this, a total of 8 groups were created. Table 1 shows the anthropometric measurement results. An imbalance was found between male and female subjects. Obese subjects were slightly older in all groups. Weight, BMI, waist circumference and WHtR were at a higher level in all obesity groups.

### 3.2 Results of isokinetic trunk strength measurements

#### 3.2.1 Absolute trunk strength

Results of the isokinetic trunk strength measurements and the *t*‐tests/Welch tests are shown in Table 2. Absolute trunk flexor strength was significantly greater in obese subjects, than non‐obese subjects, in both the BMI and age‐standardized WHtR groups (Table 2). Absolute trunk extensor strength was higher in obese subjects in 2 of 4 groups (discriminated by BMI and age‐standardized WHtR) (Table 2). For the other 2 groups, trunk extensor strength was actually lower in obese subjects (discriminated by waist circumference and WHtR) (Table 2).

**TABLE 2:** Isokinetic trunk strength measurements in the groups with or without obesity.

Parameter	BMI normal (*n* = 2890)	BMI pathological (*n* = 290)	*t*‐test	WC normal (*n* = 2769)	WC pathological (*n* = 359)	*t*‐test	WHtR normal (*n* = 2711)	WHtR pathological (*n* = 417)	*t*‐test	Age‐related WHtR normal (*n* = 1778)	Age‐related WHtR pathological (*n* = 649)	*t*‐test
FPT (Nm)	228.2	252.53	<0.001	230.29	231.18	0.529	230.41	231.09	0.811	224.41	245.44	<0.001
±SD	(±51.33)	(±58.89)	**	(±52.18)	(±56.01)	*	(±52.42)	(±54.05)	*	(±52.22)	(±54.96)	*
EPT (Nm)	400.66	443.16	<0.001	405.96	397.92	0.168	407.14	391.38	0.007	392.55	437.88	<0.001
±SD	(±102.90)	(±106.76)	*	(±101.68)	(±121.52)	**	(±102.79)	(±111.78)	**	(±107.37)	(±105.96)	*
FPT/BW (Nm/kg)	2.73	2.35	<0.001	2.75	2.22	<0.001	2.75	2.29	<0.001	2.72	2.63	<0.001
±SD	(±0.53)	(±0.56)	*	(±0.52)	(±0.49)	*	(±0.52)	(±0.51)	*	(±0.56)	(±0.56)	*
EPT/BW (Nm/kg)	4.79	4.11	<0.001	4.85	3.77	<0.001	4.86	3.85	<0.001	4.76	4.68	0.17
±SD	(±1.09)	(±0.94)	**	(±1.04)	(±0.97)	*	(±1.05)	(±0.95)	*	(±1.18)	(±1.06)	**

*Note*: Age related WtHR pathological = age 18–39 years ≥0.50, 40–49 years ≥0.55, ≥50 years ≥0.60; age‐related WtHR normal = age 18–39 years <0.50, 40–49 years <0.55, ≥50 years <0.60; BMI normal = <30 kg/m^2^; BMI pathological= > 30 kg/m^2^; *t*‐test = significance of *t*‐test is displayed to demonstrate significant group differences between groups with or without obesity (*in case of variance homogeneity *t*‐test was used, **in case of variance heterogeneity welch‐test was used); WC normal = <102 cm in men and <88 cm in women; WC pathological = ≥102 cm in men and ≥88 cm in women; WHtR normal = <0.55; WHtR pathological = ≥0.55.

Abbreviations: BMI, body mass index; SD, standard deviation; WtHR, waist‐to‐height ratio; WC, waist circumference.

#### 3.2.2 Body weight‐adapted trunk strength

Lower body weight‐adapted trunk flexor and trunk extensor strength was detected in obese subjects in all groups (Table 2).

### 3.3 Relationship between trunk strength and anthropometric parameters

#### 3.3.1 Absolute trunk strength

**FIGURE 1:**
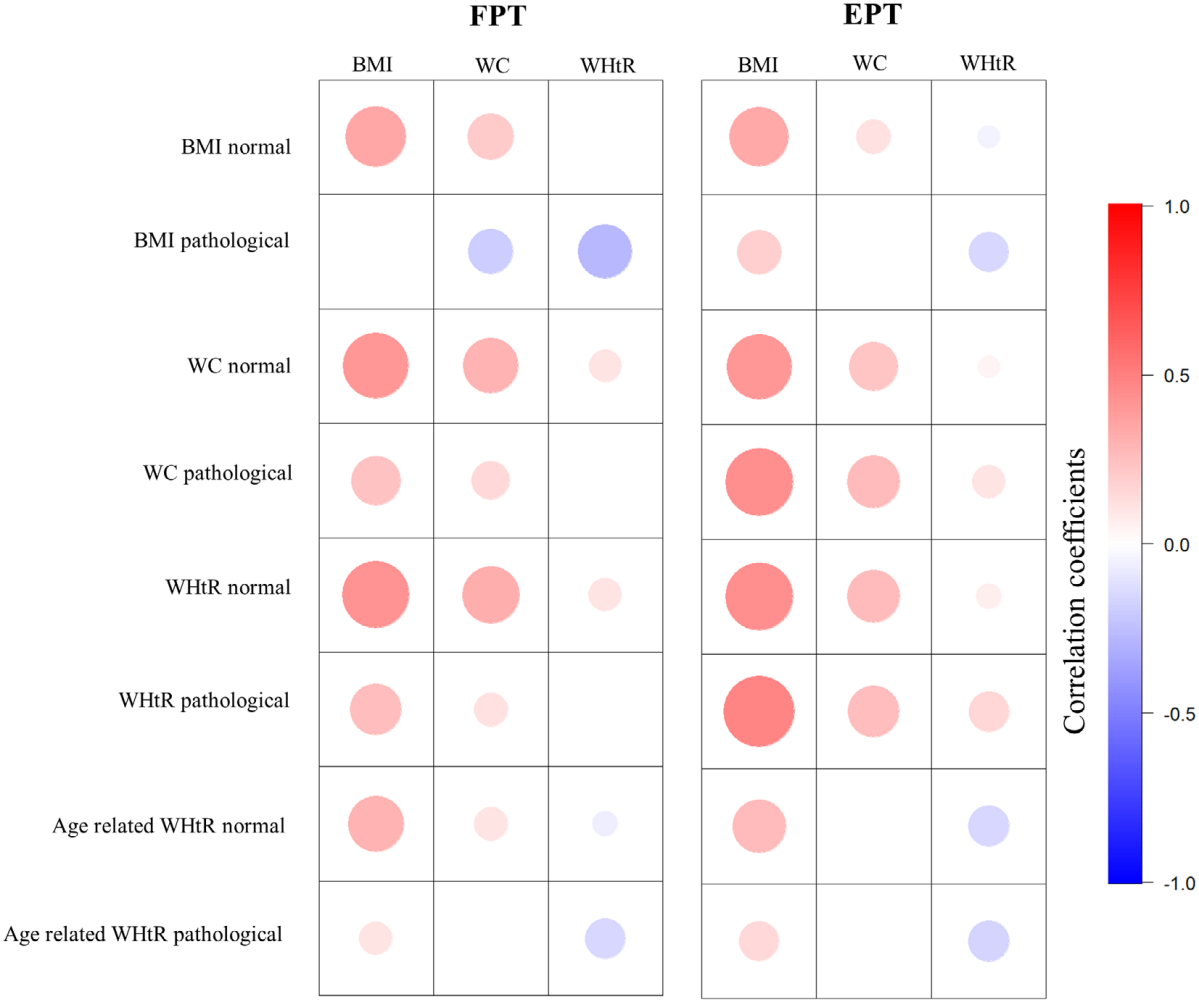
Correlogram of absolute trunk strength with body mass index, waist circumference, and waist‐to‐height ratio in the different subgroups divided by BMI, WC, WHtR, and age‐related WHtR. Age‐related WtHR pathological = age 18–39 years ≥0.50, 40–49 years ≥0.55, ≥50 years ≥0.60; age‐related WtHR normal = age 18–39 years <0.50, 40–49 years <0.55, ≥50 years <0.60; BMI normal = <30 kg/m^2^; WC normal = <102 cm in men and <88 cm in women; WC pathological = ≥102 cm in men and ≥88 cm in women; WtHR, waist‐to‐height‐ratio; WHtR normal = <0.55; WHtR pathological = ≥0.55; BMI pathological ≥ 30 kg/m^2^; BMI, body mass index; EPT, extension peak torque; FPT, flexion peak torque; WC, waist circumference.

Correlation coefficients between absolute trunk strength parameters and BMI, waist circumference and WHtR were positive in almost all groups of non‐obese subjects (Figure 1 and Table S1). In contrast, the correlation coefficients in obese subjects were, with a few exceptions, weaker positive, stronger negative or even sign‐inverted compared to the non‐obese subjects (Figure 1 and Table S1). Mixed linear regression analyses showed weaker positive regression coefficients for the variables BMI and waist circumference and weaker positive as well as more negative regression coefficients for WHtR in obese subjects (Table S3).

Accordingly, for both subjects with or without obesity, an increase in BMI and waist circumference was associated with an increase in absolute trunk flexor and extensor strength, while an increase in WHtR was associated with a decrease in absolute trunk flexor and extensor strength.

#### 3.3.2 Body weight‐adapted trunk strength

**FIGURE 2:**
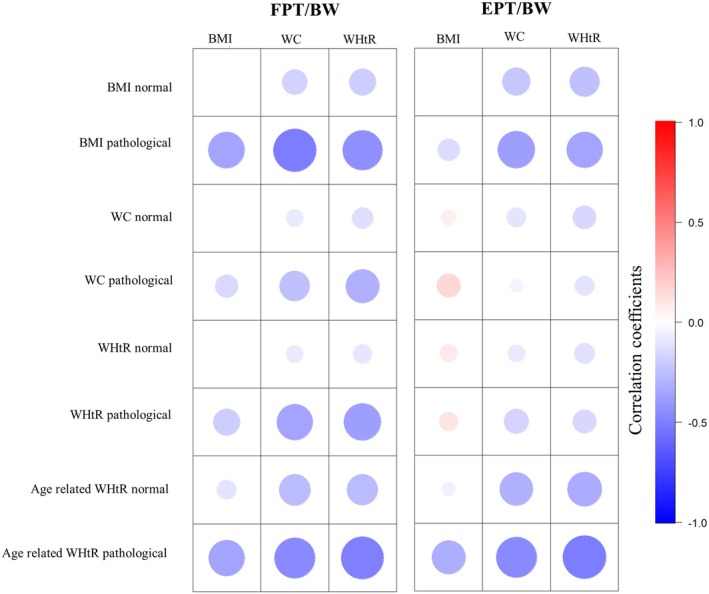
Correlogram of body weight‐adapted trunk strength with body mass index, waist circumference, and waist‐to‐height ratio in the different subgroups divided by BMI, WC, WHtR, and age‐related WHtR. Age‐related WtHR pathological = age 18–39 years ≥0.50, 40–49 years ≥0.55, ≥50 years ≥0.60; age‐related WtHR normal = age 18–39 years <0.50, 40–49 years <0.55, ≥50 years <0.60; BMI normal = <30 kg/m^2^; BMI pathological= > 30 kg/m^2^; FPT/BW = body weight‐adapted trunk flexion peak torque; EPT/BW = body weight‐adapted trunk extension peak torque; WC normal = <102 cm in men and <88 cm in women; WC pathological = ≥102 cm in men and ≥88 cm in women; WHtR normal = <0.55; WHtR pathological = ≥0.55; BMI, body mass index; WC, waist circumference; WtHR, waist‐to‐height ratio.

BMI, waist circumference and WHtR, when analysed together with body weight‐adapted isokinetic trunk strength, showed predominantly negative correlation coefficients in non‐obese subjects (Figure 2 and Table S2). In obese subjects, correlation coefficients were stronger negative in almost all cases, except for the correlation between BMI and body weight‐adapted trunk extension strength, which showed a stronger positive coefficient (Figure 2 and Table S2).

Mixed linear regression analyses demonstrated weaker positive regression coefficients for obese subjects when considering the regressor BMI, both weaker and stronger negative regression coefficients for the regressor WHtR, while the regressor waist circumference showed no significant regression coefficients for both obese and non‐obese subjects (Table S4).

Therefore, a further increase of WHtR in obese people results in a quantitatively stronger decrease of body weight‐adapted trunk flexion strength, than in non‐obese people. In contrast, further increases of the WHtR in 3 of the 4 obesity groups resulted in a smaller decrease of trunk extensor strength than in the corresponding groups without obesity.

### 3.4 Results of non‐linear regression analyses

**FIGURE 3:**
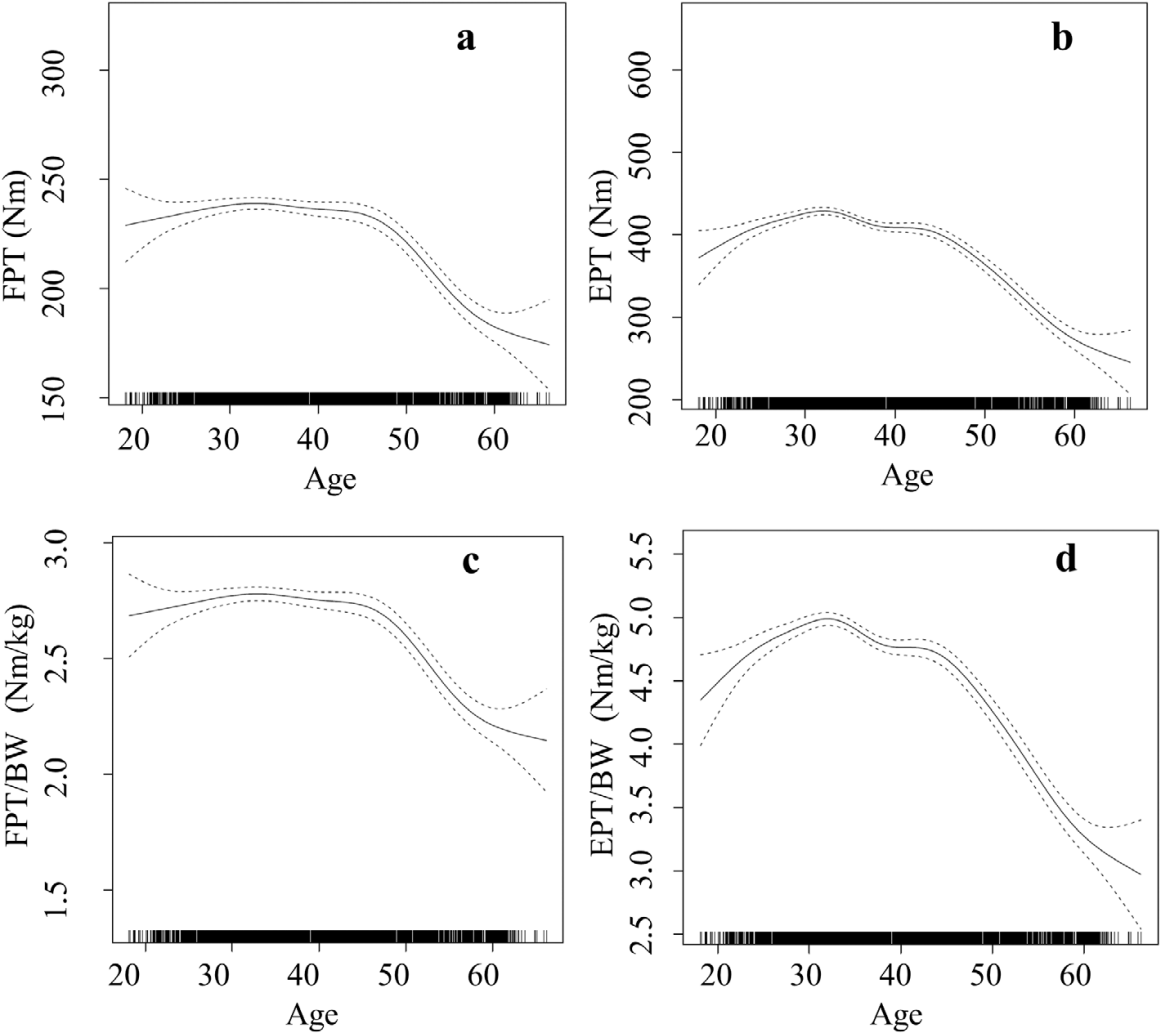
Nonlinear (gamm4) regression analyses between age [years]: (A) FPT = flexion peak torque [Nm] (*p* < .001, edf = 5.931); (B) EPT = extension peak torque [Nm] (*p* < .001, edf = 7.097); (C) FPT/BW = body weight‐adapted flexion peak torque [Nm/kg] (*p* < .001, edf = 5.841); (D) EPT/BW = body weight‐adapted extension peak torque [Nm/kg] (*p* < .001, edf = 7.093). edf, effective degrees of freedom; *p*, level of significance; *n*, 3128.

**FIGURE 4:**
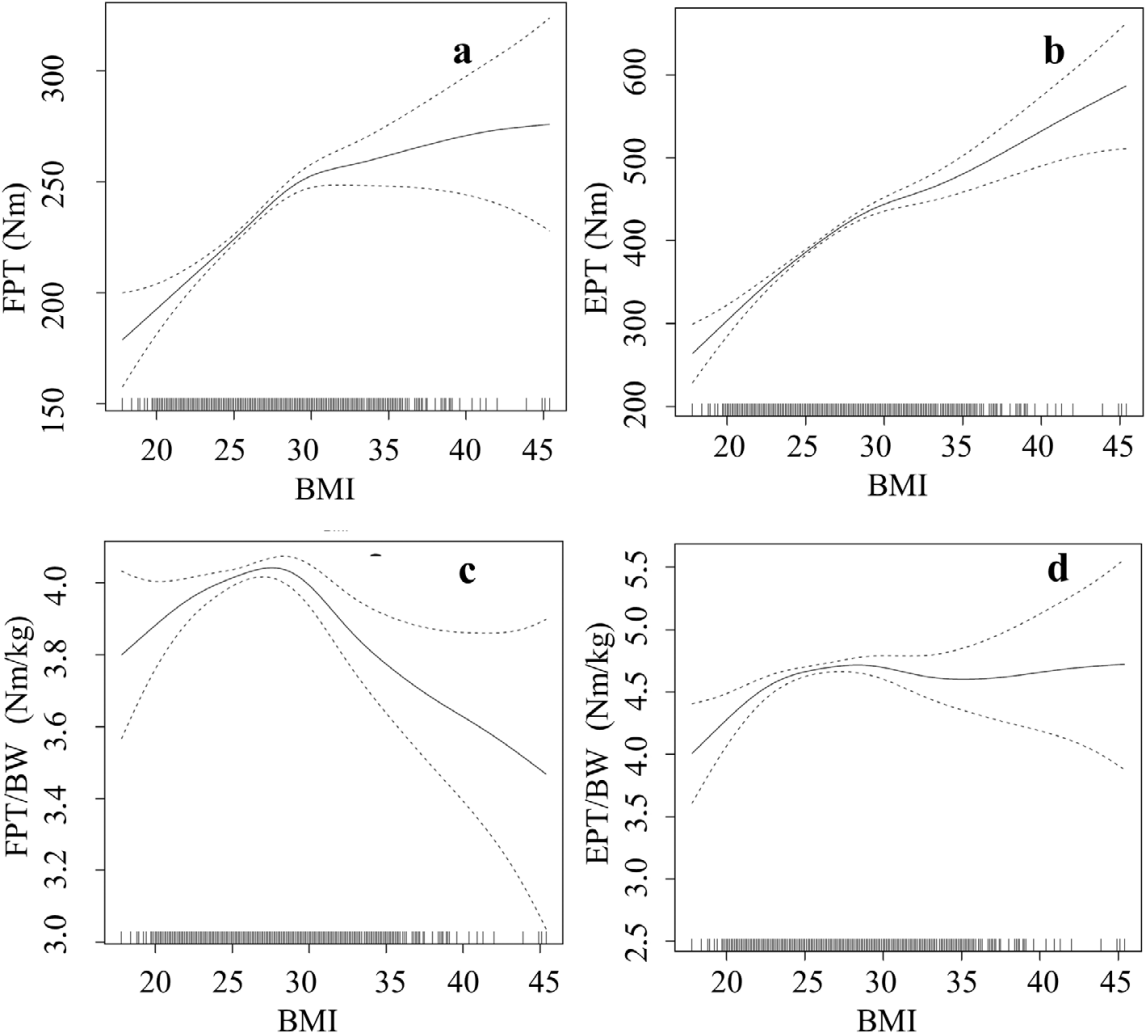
Nonlinear (gamm4) regression analyses between BMI = body mass index [kg/m^2^] and (A) FPT = flexion peak torque [Nm] (*p* < .001, edf = 3.862), (B) EPT = extension peak torque [Nm] (*p* < .001, edf = 4.005), (C): FPT/BW = body weight‐adapted flexion peak torque [Nm/kg] (*p* < .001, edf = 6.042), (D) EPT/BW = body weight‐adapted extension peak torque [Nm/kg] (*p* < .001, edf = 4.118). edf, effective degrees of freedom; *p*, level of significance; *n* = 3128 for 4c following regression formula was used: FPTBW~s(BMI) + Age + WC + WHtR.

**FIGURE 5:**
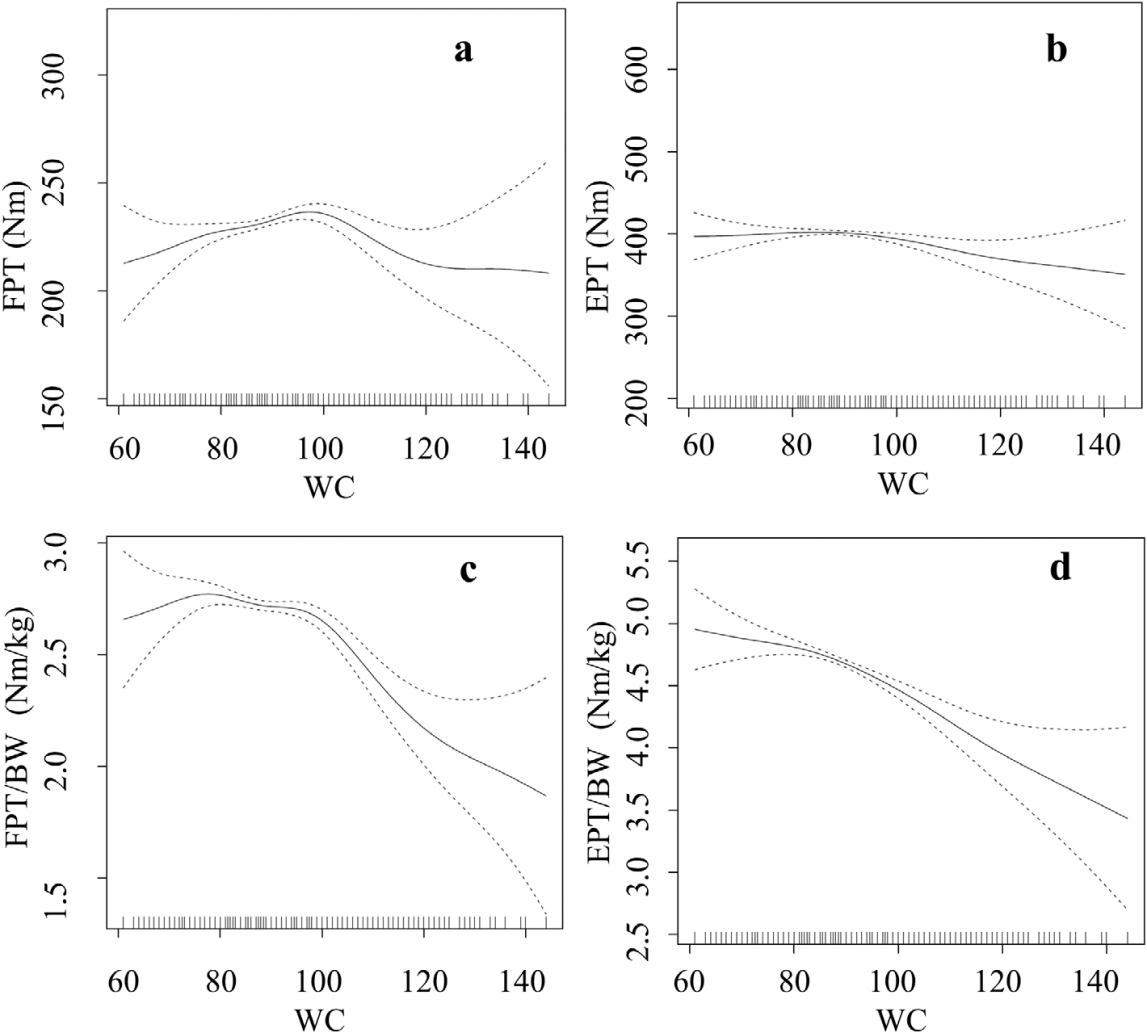
Nonlinear (gamm4) regression analyses between WC = waist circumference [cm] and (A) FPT = flexion peak torque [Nm] (*p* = .002, edf = 4.730), (B) EPT = extension peak torque [Nm] (*p* = .049, edf = 2.943), (C) FPT/BW = body weight‐adapted flexion peak torque [Nm/kg] (*p* < .001, edf = 5.041), (D) EPT/BW = body weight‐adapted extension peak torque [Nm/kg] (*p* < .001, edf = 2.946). edf, effective degrees of freedom; *p*, level of significance; *n* = 3128.

**FIGURE 6:**
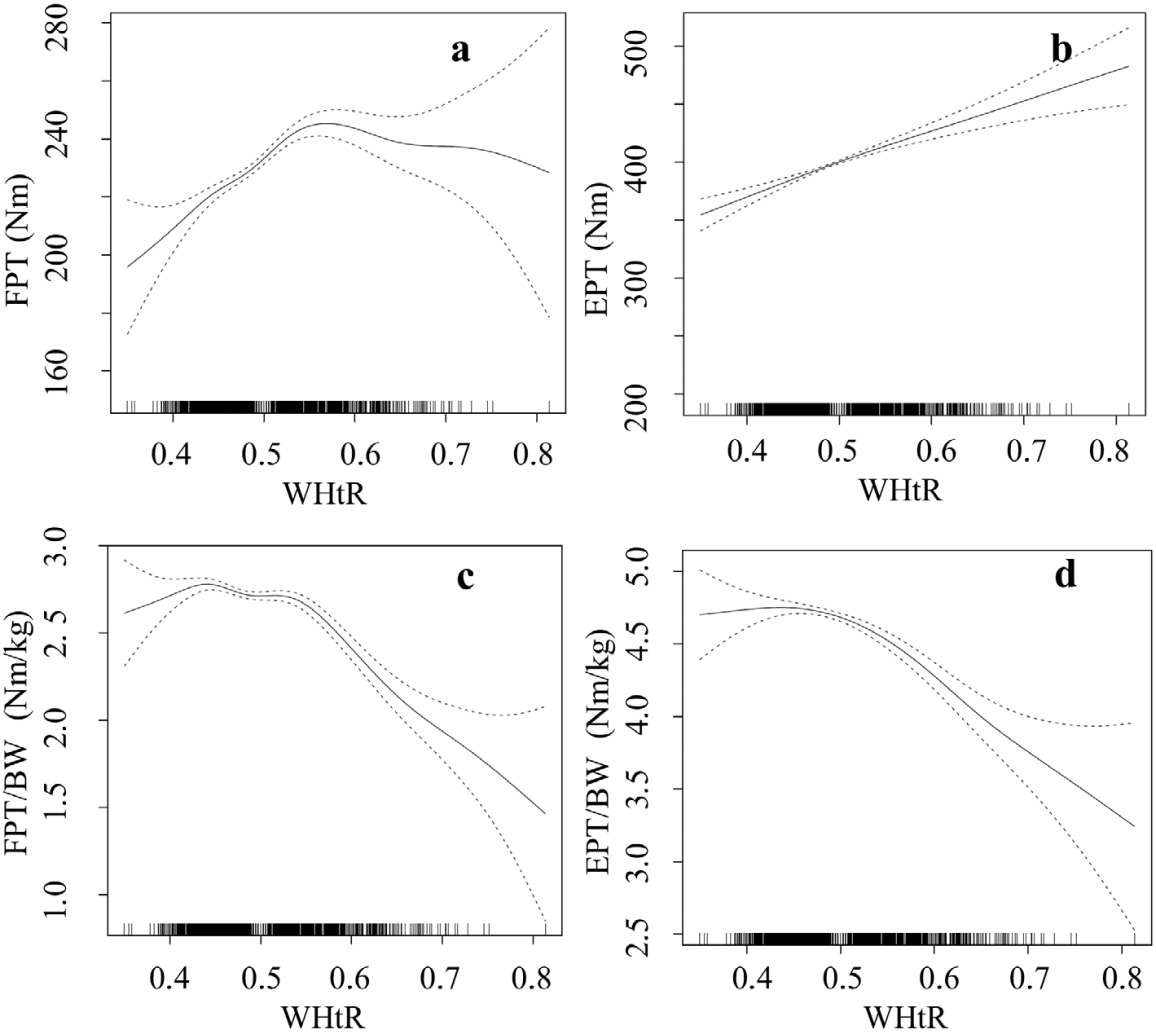
Nonlinear (gamm4) regression analyses using between WHtR = waist‐to‐height ratio and (A) FPT = flexion peak torque [Nm] (*p* < .001, edf = 4.072), (B) EPT = extension peak torque [Nm] (*p* < .001, edf = 1.525), (C) FPT/BW = body weight‐adapted flexion peak torque [Nm/kg] (*p* < .001, edf = 5.790), (D) EPT/BW = body weight‐adapted extension peak torque [Nm/kg] (*p* < .001, edf = 3.620). edf, effective degrees of freedom; *p*, level of significance; *n* = 3128.

Nearly all models demonstrated significant non‐linear relationships between trunk strength on the one hand and age, BMI, waist circumference and WHtR on the other hand (Figures 3, 4, 5, 6).

Non‐linear models explained 29.74% (resp. 21.08% for the regressor WHtR) of the variance in absolute trunk flexor strength and 44.54% (resp. 36.58% for the regressor WHtR) of the variance in absolute trunk extensor strength. However, the non‐linear models explained 24.99% (resp. 24.86% for the regressor WHtR) of the variance of the body weight‐adapted trunk flexor strength and 43.09% (resp. 42.34% for the regressor WHtR) of the variance of the body weight‐adapted trunk extensor strength.

## 4 DISCUSSION

Previous studies reported that higher BMI correlated with greater absolute trunk strength in normal and overweight people.^16^, ^20^ By separating groups in obesity/non‐obesity using BMI, an increase in BMI, waist circumference or WHtR was shown to correlate with an increase in body weight‐adapted trunk extensor and trunk flexor strength in normal‐weight subjects and with a decrease in body weight‐adapted trunk extensor and trunk flexor strength in obese subjects. This is consistent with previous studies, too.^14^, ^15^ Potential explanations for this are training effects resulting from the increased shear stress and compression forces on the lumbar spine due to obesity.^9^, ^20^

The non‐linear regressions performed in this study for the first confirm the presence of a BMI cut‐off value of 28 kg/m^2^ for the development of both absolute and body weight‐adapted trunk strength resulting from weight gain. The well‐known, slightly higher BMI cut‐off value of 30 kg/m^2^ for the diagnosis of obesity is therefore not only suitable for indicating the increased risk of cardiovascular disease but also for indicating the adverse effect of obesity on trunk flexor and extensor strength. In the context of obesity intervention programs, a reduction in absolute trunk strength may be anticipated initially, along with a reduction in body weight and, in particular, in BMI values of approximately 28–30 kg/m^2^. The adaptation of body weight can help to avoid misleading conclusions concerning inadequate trunk strength training during this intervention.

In individuals with severe obesity, the increase in absolute trunk strength with increasing weight is relatively insignificant, as the flattening curves of the nonlinear regressions demonstrate. In contrast, there is a significant reduction in body‐weight‐adapted trunk strength. Further increase in absolute trunk strength appears to be the result of the necessity to cope with an increase in body weight. This seems to be the primary stimulus for further increases in absolute trunk strength. Body weight‐adapted trunk strength is reduced due to the limited extent of increasing absolute trunk strength in relation to the disproportionate increase of body weight. These findings correspond with those of Gadducci et al., who observed no significant differences in absolute trunk strength but significant differences in body weight‐adapted trunk strength between two groups with a median BMI of 44.7 and 53.3 kg/m^2^.^36^ Therefore, the primary factor influencing the changes observed in individuals with severe obesity is not trunk strength itself, but rather body weight. This results in a notable reduction in body weight‐adapted trunk strength.

Increased waist circumference correlated in nearly all subjects with increased absolute and decreased body weight‐adapted trunk strength. The results of the present study support the findings of Fogelholm^21^ regarding the inferior performance of trunk extensor tests in subjects, both qualitatively and quantitatively. Further, the decline in performance observed above a waist circumference of 98 cm, postulated by Michaelides,^22^ was also confirmed. Waist circumference is a more suitable measure for discriminating trunk flexor changes than trunk extensor changes, as the form of the non‐linear relationship is more parabolic in flexor muscles, as it is in extensors. A possible explanation might be that obese individuals may have increased requirements for extensor muscles due to a shift in the centre of mass to the anterior‐inferior position (^10^, ^37^). This is visible by the observed change in flexion/extension ratios with increasing obesity, which demonstrates a tendency towards greater reliance on extensor muscles.^38^ The so‐called ‘flexion pattern’ is known in scientific reports about chronic low back pain, which is based on an inability to activate the trunk flexors and leads to segmental instability.^39^ Based on the relationships shown, an increased risk for this low back pain pattern can be expected in obese people.

An increase in WHtR correlated with a decrease in absolute and body weight‐adapted trunk flexor strength in both normal‐weight and overweight subjects. However, in the non‐linear models, an increasing trunk strength (excluding absolute trunk extensor strength) was observed up to a value of 0.48 and a decrease in trunk strength beyond 0.55. On the one hand, with regard to effect sizes, WHtR was the quantitatively strongest factor, followed by BMI. This emphasizes the findings of Park et al.^13^ However, this strong negative correlation only applies to body weight‐adapted trunk strength from the WHtR level above 0.55. Therefore, the WHtR‐threshold of 0.55 should be considered when differentiation of trunk strength is necessary. But on the other hand, *t*‐tests/Welch tests as well as correlation/regression analyses described BMI as the most suitable parameter for discriminating between people with and without obesity when assessing trunk strength differences. A consistent limit or threshold value of 28 kg/m^2^ was found in both absolute and body weight‐adapted trunk strength. While waist circumference and WHtR are suitable as well, they are inconsistent for absolute trunk extensor strength. In consequence, we recommend BMI to assess changes in trunk strength.

The main limitation of this study was the fact that the study population contains predominantly young, male subjects. Women and people with severe obesity were under‐represented within this sample of military personnel. The visible difference in the direction of the regression coefficients and correlation coefficients may be explained by auto‐correlation, but primarily by the (i.e., parabolic) non‐linearity of the relationships.

Regression and correlation analyses can generate predictive insights, though causality cannot be inferred. While BMI, waist circumference and WHtR showed weak to moderate associations with obesity‐related physical and psychological changes, causal links to back pain and subsequently to reduced trunk strength have predominantly been observed in populations with severe obesity (^7^, ^8^, ^9^, ^10^, ^11^, ^12^, ^40^, ^41^, ^42^, ^43^).

Elevated waist circumference is linked to a higher risk of both acute and chronic back pain.^40^ However, no causal relationship has been established between absolute trunk strength and back pain up to date.^16^ Although patients with back pain exhibit weaker weight‐adjusted trunk strength, no significant differences were identified across groups.^19^ Regarding these findings, implementing weight‐adjusted trunk strength assessment in obese patients with back pain is clinically appropriate and should become a routine component of future studies.

Trunk muscle training positively affects pain intensity and quality of life in back pain patients by improving mobility, reducing obesity‐related inflammatory mediators and stabilizing the mental well‐being of patients (^7^, ^44^). Normalizing BMI, waist circumference and WHtR may initially lead to reductions in absolute trunk strength. To distinguish decreases due to weight loss from those due to insufficient trunk muscle training, weight‐adjusted trunk strength should be assessed. Further studies on obesity intervention are necessary to support these findings.

With low compliance in back pain obese patients, not only individual exercise plans with regular follow‐up examinations, such as isokinetic trunk strength measurements, are necessary,^7^ but also strategies to increase patient adherence. Specifically, measuring body weight‐adjusted trunk strength can support adherence by providing tangible evidence of progress during regular follow‐ups, reinforcing training success, giving biofeedback and motivating sustained engagement.

## 5 CONCLUSION

For BMI, age and WHtR, non‐linear relationships with absolute trunk strength were found. When considering body weight‐adapted trunk strength, non‐linear relationships were found for the smoothed regressors age, BMI, waist circumference and WHtR.

In particular, waist circumference and WHtR have a strong negative coherence with body weight‐adapted trunk strength in obese subjects, while there is a weaker negative correlation in normal‐weight patients. In the context of obesity intervention programs, a decrease in absolute trunk strength may initially be expected along with a decrease in body weight. To avoid misleading conclusions concerning inadequate trunk strength training during this intervention, the body weight‐adapted trunk strength should be considered. To assess changes in trunk strength during weight loss, it is recommended that BMI is used as a discrimination parameter, as indicated by the results of the *t*‐test and correlation/regression analyses. It should be noted that trunk strength decreases below a threshold of 28–30 kg/m^2^ (absolute and body‐weight adapted), which is a key finding of this study.

Measuring body weight‐adapted trunk strength may also be helpful as a biofeedback measure for the regular monitoring of progress and success. Prospective studies should further investigate the influence of total body fat percentage on trunk strength instead of the indices mentioned here, while also taking gender into account.

## AUTHOR CONTRIBUTIONS

*Conceptualization and methodology*: Daniel Geissler, Andreas Lison, and Christoph Schulze. *Data curation*: Daniel Geissler, Andreas Lison, and Christoph Schulze. *Formal analysis and investigation, verification, and visualization*: Daniel Geissler. *Writing ‐ original draft preparation*: Daniel Geissler and Christoph Schulze. *Writing – review and editing*: Daniel Geissler, Andreas Lison, and Christoph Schulze. *Resources*: Andreas Lison and Christoph Schulze. *Supervision*: Christoph Schulze. All authors have read the final version of this article, approved it, and agreed with the order of authorship. All listed authors therefore met the ICMJE recommendations of authorship.

## FUNDING INFORMATION

The authors did not receive support from any organization for the submitted work.

## CONFLICT OF INTEREST STATEMENT

The authors have no financial or non‐financial interests to disclose.

## Supporting information


**Table S1.** Pearson correlation coefficients of BMI, waist circumference, and waist‐to‐height ratio with trunk flexion/extension strength in the groups with or without obesity.
**Table S2.** Pearson correlation of BMI, waist circumference, and waist‐to‐height ratio with body weight adapted trunk flexion/extension strength in the groups with or without obesity.
**Table S3.** Mixed linear regression analyses of BMI, waist circumference, and waist‐to‐height ratio with trunk flexion/extension strength in the groups with or without obesity.
**Table S4.** Mixed linear regression analyses of BMI, waist circumference, and waist‐to‐height ratio with body weight adapted trunk flexion/extension strength in the groups with or without obesity.

## Data Availability

The datasets used and/or analysed during the current study are available from the corresponding author on reasonable request.
