# Supplementary material for: Relevance of body weight adaptation and modern obesity‐defining parameters in the analysis of isokinetic trunk strength in people with obesity – A retrospective analysis
Source: Clin Obes. 2025 Jan 23;15(3):e12736. doi: 10.1111/cob.12736 (PMC12096046; doi:10.1111/cob.12736)
Supplement: Supplementary file 1 — Table S1. Pearson correlation coefficients of BMI, waist circumference, and waist‐to‐height ratio with trunk flexion/extension strength in the groups with or without obesity. Table S2. Pearson correlation of BMI, waist circumference, and waist‐to‐height ratio with body weight adapted trunk flexion/extension strength in the groups with or without obesity. Table S3. Mixed linear regression analyses of BMI, waist circumference, and waist‐to‐height ratio with trunk flexion/extension strength in the groups with or without obesity. Table S4. Mixed linear regression analyses of BMI, waist circumference, and waist‐to‐height ratio with body weight adapted trunk flexion/extension strength in the groups with or without obesity. [file COB-15-e12736-s001.pdf]

Relevance of body weight adaptation and modern obesity-defining parameters in the analysis of isokinetic trunk strength in people with obesity- a retrospective analysis

-SUPPLEMENTARY MATERIAL-

Authors:

Daniel Geissler [1], Andreas Lison [2], Christoph Schulze [1, 2,3]

1 Orthopädische Klinik und Poliklinik, Doberaner Str. 142, D-18057 Rostock, Germany.

2 Zentrum für Sportmedizin der Bundeswehr, Dr.-Rau-Allee 32, D-48231 Warendorf, Germany.

3 Universitätsinstitut für Physikalische Medizin und Rehabilitation, Universitätsklinikum Salzburg Salzburger Landeskliniken, Müllner Hauptstr. 48
A-5020 Salzburg, Austria.

Corresponding Author:

Daniel Geissler
Orthopädische Klinik und Poliklinik, Universitätsmedizin Rostock,
Doberaner Str. 142, D-18057 Rostock, Germany
E-Mail: daniel.geissler@uni-rostock.de
Tel.: +49(0)381-494-9379
Fax: +49(0)381-494-9349

Supplemental table 1 Pearson correlation coefficients of BMI, waist circumference- and Waist-to-Height-Ratio with trunk flexion/extension strength in the groups with or without obesity.

	FPT			*EPT*		
group	*BMI*	*WC*	*WHtR*	*BMI*	*WC*	*WHtR*
BMI normal	0,349 **	0,202 **	0,024	0,330 **	0,113 **	-0,05 *
p-value	*(<0.001)*	*(<0.001)*	*(0.196)*	*(<0.001)*	*(<0.001)*	*(0.008)*
BMI pathological	-0,078	-0,193 **	-0,279 **	0,189 **	-0,019	-0,152 *
p-value	*(0.183)*	*(<0.001)*	*(<0.001)*	*(<0.001)*	*(0.744)*	*(0.009)*
WC normal	0,408 **	0,290 **	0,101 **	0,402 **	0,227 **	0,049 *
p-value	*(<0.001)*	*(<0.001)*	*(<0.001)*	*(<0.001)*	*(<0.001)*	*(0.01)*
WC pathological	0,231 **	0,143 *	-0,051	0,436 **	0,265 **	0,106 *
p-value	*(<0.001)*	*(<0.001)*	*(0.381)*	*(<0.001)*	*(<0.001)*	*(0.044)*
WHtR normal	0,424 **	0,311 **	0,105 **	0,432 **	0,264 **	0,064 **
p-value	*(<0.001)*	*(<0.001)*	*(<0.001)*	*(<0.001)*	*(<0.001)*	*(<0.001)*
WHtR pathological	0,254 **	0,110 **	-0,037	0,478 **	0,252 **	0,158 **
p-value	*(<0.001)*	*(<0.001)*	*(0.445)*	*(<0.001)*	*(<0.001)*	*(<0.001)*
Age related WHtR normal	0,296 **	0,109 **	-0,063 *	0,267 **	0,005	-0,159 **
p-value	*(<0.001)*	*(<0.001)*	*(0.008)*	*(<0.001)*	*(0.850)*	*(<0.001)*
Age related WHtR pathological	0,108 *	0,013	-0,158 **	0,149 **	0,01	-0,161 **
p-value	*(0.006)*	*(0.748)*	*(<0.001)*	*(<0.001)*	*(0.798)*	*(<0.001)*

Age related WtHR pathological= age 18–39 years ≥ 0.50, 40–49 years ≥ 0.55, ≥50 years ≥ 0.60; **Age related WtHR normal=**age 18–39 years <0.50, 40–49 years <0.55, ≥50 years <0.60; **BMI**= Body-Mass-Index; **BMI normal**= < 30kg/m²; **BMI pathological**= > 30 kg/m²; **FPT**= flexion peak torque; **EPT**= extension peak torque; **WC=**waist circumference; **WC normal**= <102 cm in men and <88cm in women; **WC pathological**=≥102 cm in men and ≥ 88cm in women; **WtHR**= Waist-to-Height-Ratio; **WHtR normal**= <0,55; **WHtR pathological=** ≥0,55; *****=p<0.05; ******= p<0.001.

Supplemental table 2 Pearson correlation of BMI, waist circumference, Waist-to-Height-Ratio with body weight adapted trunk flexion/extension strength in the groups with or without obesity.

	FPT/BW			*EPT/BW*		
group	*BMI*	*WC*	*WHtR*	*BMI*	*WC*	*WHtR*
BMI normal	0,036	-0,177**	-0,197 **	0,008	-0,212 **	-0,242 **
p-value	*(0.056)*	*(<0.001)*	*(<0.001)*	*(0.683)*	*(<0.001)*	*(<0.001)*
BMI pathological	-0,36	-0,505 **	-0,433 **	-0,135 *	-0,38	-0,352 *
p-value	*(<0.001))*	*(<0.001)*	*(<0.001)*	*(0.02)*	*(<0.001)*	*(<0.001)*
WC normal	0,021	-0,088	-0,127 **	0,067 **	-0,108 **	-0,152**
p-value	*(0.269)*	*(<0.001)*	*(<0.001)*	*(<0.001)*	*(<0.001)*	*(<0.001)*
WC pathological	-0,148 *	-0,249 **	-0,310 **	0,157 *	-0,049	-0,11 *
p-value	*(0.005)*	*(<0.001)*	*(<0.001)*	*(0.003)*	*(0.350)*	*(0.037)*
WHtR normal	0,024	-0,087 **	-0,1 **	0,089 **	-0,087 **	-0,12 **
p-value	*(0.209)*	*(<0.001)*	*(<0.001)*	*(<0.001)*	*(<0.001)*	*(<0.001)*
WHtR pathological	-0,198 **	-0,357 **	-0,376 **	0,101 *	-0,169 *	-0,155 *
p-value	*(<0.001)*	*(<0.001)*	*(<0.001)*	*(0.04)*	*(0.001)*	*(0.001)*
Age related WHtR normal	-0,106 **	-0,269 **	-0,266 **	-0,056 *	-0,303 **	-0,326 **
p-value	*(<0.001)*	*(<0.001)*	*(<0.001)*	*(0.018)*	*(<0.001)*	*(<0.001)*
Age related WHtR pathological	-0,356 **	-0,451 **	-0,495 **	-0,312 **	-0,452 **	-0,507 **
p-value	*(<0.001)*	*(<0.001)*	*(<0.001)*	*(<0.001)*	*(<0.001)*	*(<0.001)*

Age related WtHR pathological= age 18–39 years ≥ 0.50, 40–49 years ≥ 0.55, ≥50 years ≥ 0.60; **Age related WtHR normal=**age 18–39 years <0.50, 40–49 years <0.55, ≥50 years <0.60; **BMI**= Body-Mass-Index; **BMI normal**= < 30kg/m²; **BMI pathological**= > 30 kg/m²; **FPT/BW**= body weight adapted trunk flexion peak torque; **EPT/BW**= body weight adapted trunk extension peak torque; **WC=**waist circumference; **WC normal**= <102 cm in men and <88cm in women; **WC pathological**=≥102 cm in men and ≥ 88cm in women; **WtHR**= Waist-to-Height-Ratio; **WHtR normal**= <0,55; **WHtR pathological=** ≥0,55; *****=p<0.05; ******= p<0.001.

Supplemental table 3 Mixed **l**inear Regression analyses of BMI, waist circumference and Waist-to-Height-Ratio with trunk flexion/extension strength in the groups with or without obesity.

	FPT			*EPT*		
group	*BMI*	*WC*	*WHtR*	*BMI*	*WC*	*WHtR*
BMI normal	13,362 **	4,86 **	-1294,65 **	31,08 **	7,662 **	-2590,18 **
p-value	*(<0.001)*	*(<0.001)*	*(<0.001)*	*(<0.001)*	*(<0.001)*	*(<0.001)*
BMI pathological	8,667**	3,282 **	-1292,545 **	26,957 **	5,577 **	-2375,624 **
p-value	*(<0.001)*	*(<0.001)*	*(<0.001)*	*(<0.001)*	*(<0.001)*	*(<0.001)*
WC normal	12,977 **	5,155 **	-1255,62 **	29,008 **	8,376 **	-2475,314 **
p-value	*(<0.001)*	*(<0.001)*	*(<0.001)*	*(<0.001)*	*(<0.001)*	*(<0.001)*
WC pathological	9,437 **	4,01 **	-1264,024 **	27,906 **	7,289 **	-2562,685 **
p-value	*(<0.001)*	*(<0.001)*	*(<0.001)*	*(<0.001)*	*(<0.001)*	*(<0.001)*
WHtR normal	13,004 **	4,991 **	-1241,148 **	29,352 **	8,162 **	-2442,195 **
p-value	*(<0.001)*	*(<0.001)*	*(<0.001)*	*(<0.001)*	*(<0.001)*	*(<0.001)*
WHtR pathological	10,126 **	3,145 **	-1275,322 **	27,241 **	4,347 **	-2140,974 **
p-value	*(<0.001)*	*(<0.001)*	*(<0.001)*	*(<0.001)*	*(<0.001)*	*(<0.001)*
Age related WHtR normal	13,023 **	4,767 **	-1436,006 **	31,408 **	7,704 **	-3020,874 **
p-value	*(<0.001)*	*(<0.001)*	*(<0.001)*	*(<0.001)*	*(<0.001)*	*(<0.001)*
Age related WHtR pathological	10,549 **	4,423 **	-1529,09 **	24,967 **	8,088 **	-3136,579 **
p-value	*(<0.001)*	*(<0.001)*	*(<0.001)*	*(<0.001)*	*(<0.001)*	*(<0.001)*

Age related WtHR pathological= age 18–39 years ≥ 0.50, 40–49 years ≥ 0.55, ≥50 years ≥ 0.60; **Age related WtHR normal=**age 18–39 years <0.50, 40–49 years <0.55, ≥50 years <0.60; **BMI**= Body-Mass-Index; **BMI normal**= < 30kg/m²; **BMI pathological**= > 30 kg/m²; **FPT**= flexion peak torque; **EPT**= extension peak torque; **WC=**waist circumference; **WC normal**= <102 cm in men and <88cm in women; **WC pathological**=≥102 cm in men and ≥ 88cm in women; **WtHR**= Waist-to-Height-Ratio; **WHtR normal**= <0,55; **WHtR pathological=** ≥0,55; *****=p<0.05; ******= p<0.001.

Supplemental table 4 Mixed **l**inear Regression analyses of BMI, waist circumference, Waist-to-Height-Ratio-with body weight adapted trunk flexion/extension strength in the groups with or without obesity.

	FPT/BW			*EPT/BW*		
group	*BMI*	*WC*	*WHtR*	*BMI*	*WC*	*WHtR*
BMI normal	0,057 **	-0,006	-4,895 **	0,193 **	-0,01	-12,362 **
p-value	*(<0.001)*	*(0.838)*	*(<0.001)*	*(<0.001)*	*(0.083)*	*(<0.001)*
BMI pathological	0,029	-0,011	-5,264 **	0,147 **	-0,02	-10,164 **
p-value	*(0.059)*	*(0.13)*	*(<0.001)*	*(<0.001)*	*(0.094)*	*(<0.001)*
WC normal	0,051 **	0,001	-4,252 **	0,167 **	0,005	-10,714 **
p-value	*(<0.001)*	*(0.673)*	*(<0.001)*	*(<0.001)*	*(0.418)*	*(<0.001)*
WC pathological	0,031 *	0,001	-5,439 **	0,163 **	0,006	-13,348 **
p-value	*(0.004)*	*(0.873)*	*(<0.001)*	*(<0.001)*	*(0.620)*	*(<0.001)*
WHtR normal	0,052 **	0,001	-4,158 **	0,170 **	-0,006	-10,193 **
p-value	*(<0.001)*	*(0.977)*	*(<0.001)*	*(<0.001)*	*(0.258)*	*(<0.001)*
WHtR pathological	0,037 **	-0,01	-5,322 **	0,157 **	-0,029 *	-8,965 **
p-value	*(<0.001)*	*(0.071)*	*(<0.001)*	*(<0.001)*	*(0.008)*	*(<0.001)*
Age related WHtR normal	0,057 **	0,001	-6,725 **	0,206 **	-0,005	-17,906 **
p-value	*(<0.001)*	*(0.946)*	*(<0.001)*	*(<0.001)*	*(0.463)*	*(<0.001)*
Age related WHtR pathological	0,029 *	-0,003	-6,831 **	0,108 **	-0,004	-16,281 **
p-value	*(0.006)*	*(0.583)*	*(<0.001)*	*(<0.001)*	*(0.634)*	*(<0.001)*

Age related WtHR pathological= age 18–39 years ≥ 0.50, 40–49 years ≥ 0.55, ≥50 years ≥ 0.60; **Age related WtHR normal=**age 18–39 years <0.50, 40–49 years <0.55, ≥50 years <0.60; **BMI**= Body-Mass-Index; **BMI normal**= < 30kg/m²; **BMI pathological**= > 30 kg/m²; **FPT/BW**= body weight adapted trunk flexion peak torque; **EPT/BW**= body weight adapted trunk extension peak torque; **WC=**waist circumference; **WC normal**= <102 cm in men and <88cm in women; **WC pathological**=≥102 cm in men and ≥ 88cm in women; **WtHR**= Waist-to-Height-Ratio; **WHtR normal**= <0,55; **WHtR pathological=** ≥0,55; *=p<0.05; **= p<0.001.
